# Exploring the transcriptional landscape of plant circadian rhythms using genome tiling arrays

**DOI:** 10.1186/gb-2009-10-2-r17

**Published:** 2009-02-11

**Authors:** Samuel P Hazen, Felix Naef, Tom Quisel, Joshua M Gendron, Huaming Chen, Joseph R Ecker, Justin O Borevitz, Steve A Kay

**Affiliations:** 1 Section of Cell and Developmental Biology, University of California San Diego, Gilman Drive, La Jolla, CA 92093-0130, USA; 2 School of Life Science, Ecole Polytechnique Federale de Lausanne (EPFL), CH-1015 Lausanne, Switzerland; 3 Plant Biology Laboratory and Genome Analysis Laboratory, The Salk Institute for Biological Studies, N. Torrey Pines Road, La Jolla, CA 92037, USA; 4 Department of Evolution and Ecology, University of Chicago, E. 57th Street, Chicago, IL 60637, USA; 5 Biology Department, University of Massachusetts, N. Pleasant Street, Amherst, MA 01003, USA

## Abstract

**Background:**

Organisms are able to anticipate changes in the daily environment with an internal oscillator know as the circadian clock. Transcription is an important mechanism in maintaining these oscillations. Here we explore, using whole genome tiling arrays, the extent of rhythmic expression patterns genome-wide, with an unbiased analysis of coding and noncoding regions of the *Arabidopsis *genome.

**Results:**

As in previous studies, we detected a circadian rhythm for approximately 25% of the protein coding genes in the genome. With an unbiased interrogation of the genome, extensive rhythmic introns were detected predominantly in phase with adjacent rhythmic exons, creating a transcript that, if translated, would be expected to produce a truncated protein. In some cases, such as the MYB transcription factor *AT2G20400*, an intron was found to exhibit a circadian rhythm while the remainder of the transcript was otherwise arrhythmic. In addition to several known noncoding transcripts, including microRNA, trans-acting short interfering RNA, and small nucleolar RNA, greater than one thousand intergenic regions were detected as circadian clock regulated, many of which have no predicted function, either coding or noncoding. Nearly 7% of the protein coding genes produced rhythmic antisense transcripts, often for genes whose sense strand was not similarly rhythmic.

**Conclusions:**

This study revealed widespread circadian clock regulation of the *Arabidopsis *genome extending well beyond the protein coding transcripts measured to date. This suggests a greater level of structural and temporal dynamics than previously known.

## Abstract 2

Whole genome tiling array analysis reveals the extent of transcriptional oscillation for both coding and non-coding genes in regulating Arabidopsis thaliana circadian rhythms

## Background

Many organisms exhibit cyclic changes in physiology and behavior in accordance with predictable changes in their daily environment, namely shifts in temperature and light intensity owing to transitioning exposure to the sun caused by the Earth's rotation. In addition to reacting directly to external stimuli, many organisms time their behavior in anticipation of periodic changes in the environment. Such circadian rhythms are believed to be adaptive and, indeed, have been demonstrated in both prokaryotic and eukaryotic photosynthetic organisms [1,2]. The endogenous timing mechanism known as circadian clocks is widespread across life and is primarily based on interlocking transcriptional feedback loops and regulated protein turnover [3].

Circadian clock regulation of transcription in plants appears to be extensive and many pathways governing processes such as photosynthesis, cold acclimation, and cell wall dynamics, for example, exhibit circadian rhythms at multiple levels [4-6]. Estimates of the extent of circadian clock regulation are primarily derived from the use of high-density oligonucleotide arrays with features that mostly correspond to the 3' end of genes annotated as protein coding (see, for example, [4-6]). Recently, there has been a flourish of transcript mapping using genome tiling arrays capable of measuring nearly all nonredundant sequences in the genome, far beyond the capability of previous studies [7-9]. In excess of the number of protein coding transcripts, noncoding RNAs (ncRNAs), which include natural antisense transcripts (NATs), appear to be a large component of the remarkably complex transcriptome in all organisms examined to date: *Arabidopsis*, *Caenorhabditis elegans*, *Chlamydomonas*, *Drosophila*, *Escheichia coli*, human, rice, and yeast [10-24]. Aside from hybridization-based detection systems, sequencing approaches such as serial analysis of gene expression (SAGE), massively parallel signature sequencing (MPSS), and directional cDNA cloning and sequencing have confirmed widespread existence of these transcripts in plants and other species [25-27]. It is not difficult to fathom the existence of numerous and sundry ncRNAs. There are several classes of long studied ncRNAs, such as transfer RNA (tRNA), ribosomal RNA (rRNA), and small nuclear RNA (snRNA) in addition to the more recently discovered small nucleolar RNA (snoRNA), microRNA (miRNA), and short interfering RNA (siRNA) [28]. Nevertheless, the existence of these specific forms does not explain the excessive ncRNAs measured by tiling arrays. This suggests a complex RNA regulatory network akin to that revealed through the study of X chromosome silencing, for example [29].

Tiling array experiments have done little to characterize large-scale transcriptional activity beyond to say it exists. Here, we explore circadian clock controlled transcriptional regulation in *Arabidopsis *using high-density oligonucleotide tiling arrays. In addition to protein coding genes and intergenic regions, we measured circadian regulation of introns, as well as clock-regulated NATs.

## Results and discussion

### Tiling array characteristics and performance

The Affymetrix *Arabidopsis *tiling arrays each contain 1,683,620 unique 25-mer oligonucleotide features. One array is composed of the forward or Watson strand and the other the reverse or Crick strand. The *Arabidopsis *Information Resource Version 7 (TAIR7) genome annotation includes a total of 32,041 genes, of which 27,029 are considered to be protein coding [30]. Nearly 95% (25,677) of the protein coding genes have at least two corresponding exon array features, as do 74% (2,863) of the transposons and pseudogenes (Table 1). Due to their small size and sequence redundancy within gene families, only 202 of the 1,123 annotated ncRNAs have at least two corresponding array features and, of those, 62 are miRNA.

**Table 1:** *Arabidopsis *genome and AtTILE1 array annotation data

Annotation	TAIR7*	AtTILE1	CCGs^†^
Protein coding	27,029	25,677	6,269
Pseudogenes or TE	3,889	2,863	81
			
Noncoding RNAs	1,123		
MicroRNA	114	62 (30)	6
Small nucleolar RNA	71	17 (29)	1
Small nuclear RNA	13	0	ND
Pre-transfer RNA	689	2 (129)	0
Ribosomal RNA	4	0	ND
Other	221	121 (29)	15
			
Total	32,041		6,372

Annotation units receiving consideration had at least two unique corresponding array features. Values in parentheses are the number of transcripts with a single corresponding feature. *The *Arabidopsis *Information Resource (TAIR) version 7 genome annotation [30]. ^†^Circadian clock regulated genes. ND, not determined; TE, transposable element.

**Figure 1:**
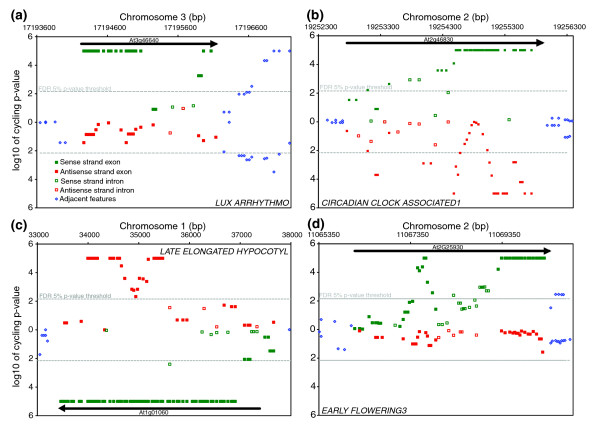
The *Arabidopsis *tiling arrays portray several interesting classes of circadian behavior in the genome. Each symbol is a feature on the tilling array showing location in the genome (x-axis) and significance of the spectral analysis (y-axis) for **(a) ***LUX ARRHYTHMO*, **(b) ***CIRCADIAN CLOCK ASSOCIATED1*, **(c) ***LATE ELONGATED HYPOCOTYL*, and **(d) ***EARLY FLOWERING3*. The top half of each panel displays the Watson strand and the bottom half the Crick strand. Individual features that exceed the false discovery rate 5% *p*-value threshold (-) are considered to have a circadian rhythm.

**Figure 2:**
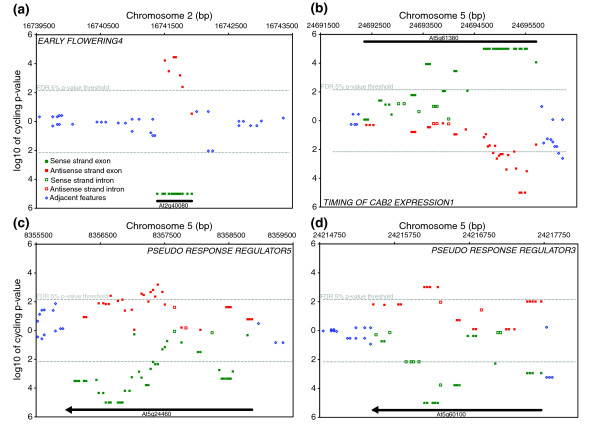
The *Arabidopsis *tiling arrays portray several interesting classes of circadian behavior in the genome. Each symbol is a feature on the tilling array showing location in the genome (x-axis) and significance of the spectral analysis (y-axis) for **(a) ***EARLY FLOWERING4*, **(b) ***TIMING OF CAB2 EXPRESSION1*, **(c) ***PSEUDO RESPONSE REGULATOR5*, and **(d) ***PSEUDO RESPONSE REGULATOR3*. The top half of each panel displays the Watson strand and the bottom half the Crick strand. Individual features that exceed the false discovery rate 5% *p*-value threshold (-) are considered to have a circadian rhythm.

**Figure 3:**
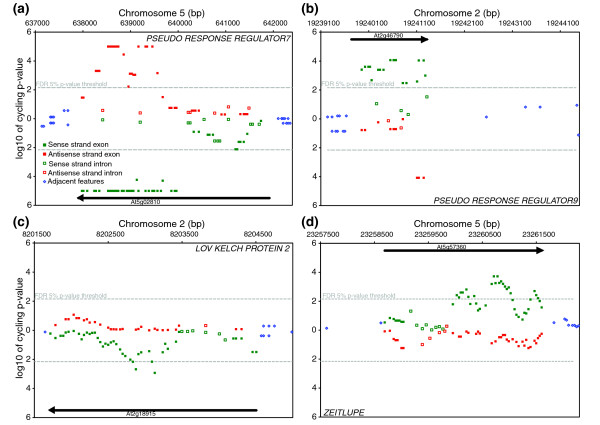
The *Arabidopsis *tiling arrays portray several interesting classes of circadian behavior in the genome. Each symbol is a feature on the tilling array showing location in the genome (x-axis) and significance of the spectral analysis (y-axis) for **(a) ***PSEUDO RESPONSE REGULATOR7*, **(b) ***PSEUDO RESPONSE REGULATOR9*, **(c) ***LOV KELCH PROTEIN2*, and **(d) ***ZEITLUPE*. The top half of each panel displays the Watson strand and the bottom half the Crick strand. Individual features that exceed the false discovery rate 5% *p*-value threshold (-) are considered to have a circadian rhythm.

**Figure 4:**
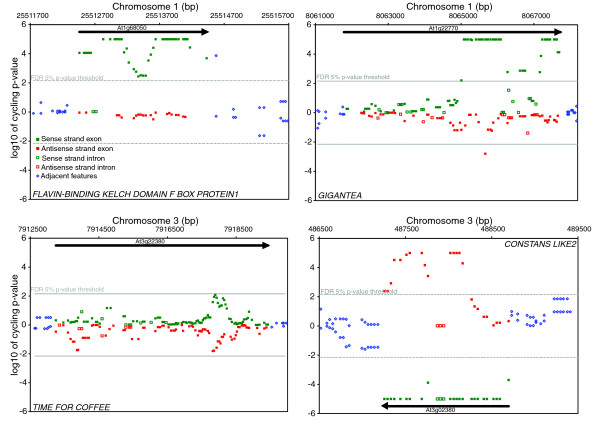
The *Arabidopsis *tiling arrays portray several interesting classes of circadian behavior in the genome. Each symbol is a feature on the tilling array showing location in the genome (x-axis) and significance of the spectral analysis (y-axis) for **(a) ***FLAVIN-BINDING KELCH DOMAIN F BOX PROTEIN1*, **(b) ***GIGANTEA*, **(c) ***TIME FOR COFFEE*, and **(d) ***CONSTANS LIKE2*. The top half of each panel displays the Watson strand and the bottom half the Crick strand. Individual features that exceed the false discovery rate 5% *p*-value threshold (-) are considered to have a circadian rhythm.

Labeled cRNA was prepared from 12 samples collected during a 2-day circadian time course at 4-hour resolution. Samples were independently hybridized to each array as previously described [4]. Spectral analysis was used to test for a circadian rhythm in the hybridization intensity of each feature across the 2-day time course. Rather than treat each feature as an independent experiment, a sliding window approach was used to exploit the redundant signal in neighboring features (see Materials and methods). As a test of the capabilities of the tiling arrays, RNA time course, and spectral analysis, we specifically looked at the expression of 14 circadian clock associated genes: *CIRCADIAN CLOCK ASSOCIATED1 *(*CCA1*), *LATE ELONGATED HYPOCOTYL *(*LHY*), *GIGANTEA *(*GI*), *TIMING OF CAB2 EXPRESSION1 *(*TOC1*), *PSEUDO RESPONSE REGULATOR3*, *5*, *7*, and *9 *(*PRR3*, *5*, *7*, and *9*), *LOV KELCH PROTEIN2 *(*LKP2*), *LUX ARRHYTHMO *(*LUX*), *EARLY FLOWERING3 *and *4 *(*ELF3 *and *ELF4*), *FLAVIN-BINDING*, *KELCH REPEAT*, *F-BOX 1 *(*FKF1*) and *ZEITLUPE *(*ZTL*) [31]. In Figures 1, 2, 3, and 4 we plot the results of the spectral analysis of the expression level time course for individual features on the array. Each of these genes had at least two exon features that satisfied the *p *< 0.005 cut-off as well as a phase (Additional data files 1 and 2) similar to that reported previously. Two clock genes with weak rhythms at the transcriptional level, *LKP2 *[32] and *ZTL*, exhibited the expected behavior (Figure 3). A clock gene that does not cycle at the transcriptional level, *TIME FOR COFFEE *[33], was similarly found not to exhibit circadian regulation (Figure 4c).

**Figure 5:**
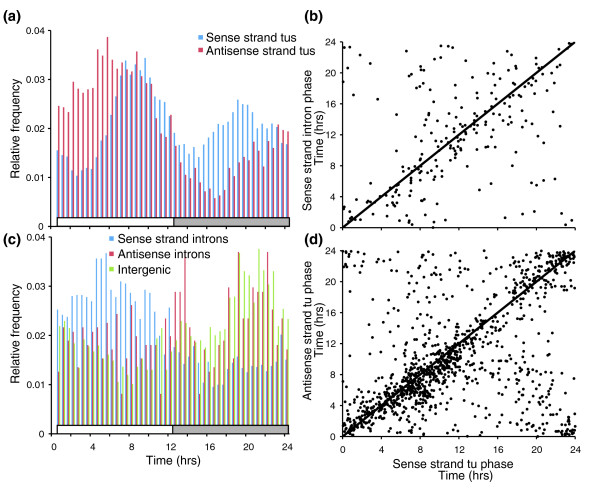
Different types of transcripts and transcription units have variable phase distributions across the day as well as within a locus. **(a) **Relative phase frequency distribution of cycling sense and antisense transcript phase. **(b) **Scatter plot of the expression phases of loci with both sense and antisense strand cycling transcripts. **(c) **Relative phase frequency distribution of cycling sense strand and antisense strand introns and intergenic transcript phase. **(d) **Scatter plot of the expression phases of transcripts and their cycling introns. tu(s), transcript unit(s).

In addition to these consistencies, we compared the tiling array dataset with a similarly produced 2-day time course [GEO:GSE8365] [34] hybridized to the Affymetrix ATH1 gene array. The spectral analysis for each gene on the gene array was plotted against all of the features for that transcript on the tilling array. While comparison between these platforms should be interpreted cautiously, there was strong accord between data sets for significance in rhythmicity as well as circadian phase (Additional data file 3). At the genome level, 24.4% of the protein coding genes were circadian clock regulated (false discovery rate < 0.05%), that is to say, the transcript exhibited a rhythmic 24-hour period over a 2-day time course (Table S3 in Additional data file 4). This result is well within the range of recent reports [35,36] that used the *Arabidopsis *ATH1 array. In these studies, more than 75% of the protein coding transcripts assayed were found to cycle when driven by various conditions of photocycles and/or thermocycles or under constant conditions. While all phases were represented, there was an increase in frequency of genes with peak expression just prior to dawn and dusk, suggesting an important role of the circadian clock in anticipating the transitions between day and night (Figure 5a). These data can also be queried and visualized at the *Arabidopsis *Cyclome Expression Database [37].

### Circadian clock regulation of introns

Unlike the design of the *Arabidopsis *ATGenome1 and ATH1 arrays, where features quantify hybridization of the sense strand transcript of the protein coding regions, AtTILE1 features also correspond to 597,856 intergenic and 301,733 intronic loci on each strand. Interestingly, these features capably detected 499 transcripts with rhythmic introns (Table S4 in Additional data file 4). In cases where cycling introns were observed in genes with cycling exons (n = 213), the introns frequently had a similar phase to the coding regions of the transcript (Figure 5b). Unlike an alternatively spliced exon, introns are nonsense sequences and their inclusion tends to introduce a translational stop, as in the examples of *ELF3 *(Figure 1b) and *CONSTANS LIKE2 *(*COL2*) (Figure 4d). Transcripts of these genes were transcriptionally verified for an exon and intron using quantitative PCR of reverse transcriptase amplified cDNA (QRT-PCR) of an experimentally independent time course (Additional data file 5). For both genes (*ELF3 *[GenBank:AY136385 and Y11994]; *COL2 *[GenBank:L81119 and L81120]), a cDNA of both splice forms, with and without the detected cycling intron, has been captured and sequenced. By assaying RNA from pooled whole seedlings with an oligonucleotide array platform, it is not clear if both variants occur in the same cell or tissue types or if they are simply immature transcripts sampled prior to complete processing. Hybridization intensities of individual features do suggest the intron variant of *COL2*, for example, is present in appreciable quantities (Additional data file 5). If so, this presents somewhat of a conundrum. For example, mutations in *ELF3 *can cause a rather dramatic effect on flowering time and circadian rhythms in *Arabidopsis *[38] and, curiously, inclusion of the second intron, as we observed, could produce a protein similar to that of the *elf3-1 *mutant [39]. In a number of instances, introns exhibited a phase differing from the coding region of the transcript by greater than 4 hours (Figure 5b).

**Figure 6:**
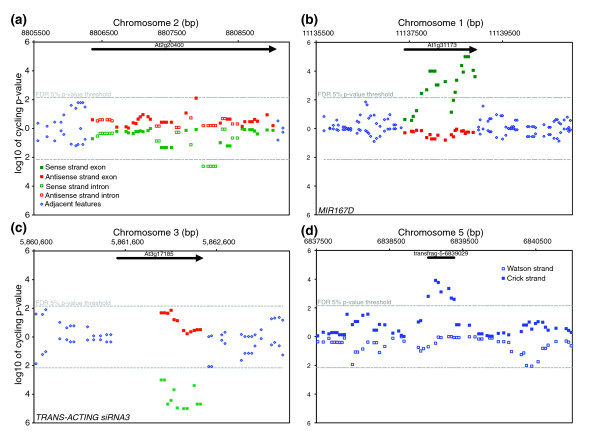
The *Arabidopsis *tiling arrays portray several interesting classes of circadian behavior in the genome. Each symbol is a feature on the tilling array showing location in the genome (x-axis) and significance of the spectral analysis (y-axis) for **(a) ***AT2G20400*, **(b) ***MIR167*, **(c) ***TRANS-ACTING siRNA3*, and **(d) **transfrag-5-6839029. The top half of each panel displays the Watson strand and the bottom half the Crick strand. Individual features that exceed the false discovery rate 5% *p*-value threshold (-) are considered to have a circadian rhythm.

Quite unexpected, 286 genes that showed no evidence of rhythmic expression of coding regions contained an intron exhibiting circadian rhythmcity (Table S5 in Additional data file 4). This form of alternative splicing or 'gated intron inclusion' could result in altered protein function that occurs at a specific time of day. For example, the fifth intron of *AT2G20400 *(Figure 6a) cycles with peak expression in the late afternoon and this was confirmed by QRT-PCR using a second experimental time course (Additional data file 5). Under these circumstances, the complete message was constitutively, or at least arrhythmically, expressed. Perhaps the point of peak rhythmic expression of the intron is a circadian clock regulated occurrence of intron inclusion where the transcribed protein is truncated. This phenomenon is not difficult to reconcile with what is known about the *Arabidopsis *genome. Among the protein coding transcripts, nearly 15% have an annotated splice variant [30], which is appreciably smaller than the proportion in mammalian genomes [40,41]. In addition to the distinction in overall proportion of splice variant genes, intron inclusion is a less frequent cause of variation in mammals but the most prevalent in *Arabidopsis*, with at least 8% of *Arabidopsis *protein coding genes exhibiting intron inclusion [42,43]. Considering that the vast proportion of the genome is diurnally and circadian regulated, including many RNA binding proteins, the occurrence of circadian gated intron inclusion is not inexplicable [35,44]. However, the exact mechanism for any one of these events and their biological relevance is not well understood. In a number of instances, the peak phase of expression of introns was observed to be 4-12 hours apart from that of the coding region of a transcript (Figure 5b).

### Circadian clock regulation of ncRNAs

Certain ncRNAs known as miRNAs fold back and form imperfect double-stranded RNAs that are processed by the Dicer and RNaseIII-like families to create approximately 22 bp fragments [45]. In plants, transcripts with exact homology to mature miRNAs are targeted for post-transcriptional regulation. Many miRNAs are responsible for silencing transcription factors associated with growth and development and their expression is often tightly regulated both developmentally and spatially [46-48]. Although the AtTILE1 arrays are capable of distinguishing only a fairly small proportion of the 114 annotated miRNAs in the *Arabidopsis *genome, several were found to cycle in 1-week-old seedlings. Our protocol amplified and is assumed to detect polyadenylated transcripts only, and in the case of the miRNA loci, some relatively large cycling premature transcripts were observed. Two miRNA in particular, *MIR160B *and *MIR167D *(Additional data file 5), target several members of the *AUXIN RESPONSE FACTOR *(*ARF*) family, members of which bind to the auxin response elements (TGTCTC) in promoters of early auxin response genes [49]. *MIR160B *targets *ARF10*, *ARF16*, and *ARF17*, which are all believed to be involved in germination and post-germination stages of growth [50,51]. *MIR167D *targets *ARF6 *and *ARF8*, which are involved in male and female reproductive development [51,52]. Two other clearly cycling miRNA are *MIR158A*, with no known target, and *MIR157A*, which targets several members of the *SQUAMOSA BINDING PROTEIN *family, *SPL3*, *SPL4*, and *SPL5*. Interestingly, the target *SPL*s and *ARF*s were not found to be circadian regulated. We speculate that for such a pattern to occur, the target must be expressed constitutively and only in cell types with rhythmic target miRNA expression. Otherwise, the signal from cells where miRNA are not expressed may obscure a rhythmic signal caused by miRNA expression in other cells. Additionally, the relationship between target degradation and miRNA concentration would need to be somewhat linear, whereas in practice it is more qualitative, requiring a certain threshold of accumulation prior to detectable degradation [53]. Therefore, the absence of a reciprocal expression pattern of the target transcripts does not rule out a specific function behind the circadian behavior of the miRNA.

The well-described complexity of *AFR *transcript regulation is also influenced by trans-acting siRNA (ta-siRNA), namely *TAS3 *[54-57]. Dicer processing of the primary *TAS *transcripts is triggered by miRNA-guided cleavage. In the case of *TAS3*, *MIR390 *directed cleavage results in a 21 bp double-stranded RNA with post-transcriptional properties similar to miRNA [58]. While both *MIR390A *(At2g38325) and *MIR390B *(At5g58465) were reliably detected by the AtTILE1 arrays, neither was found to exhibit a circadian rhythm (Additional data files 1 and 2). On the other hand, the abundance of the primary *TAS3 *transcript is clearly circadian clock regulated, a pattern confirmed in two independent time courses (Additional data file 5). While transcript abundance of *TAS3 *and possibly *TAS2 *(Additional data files 1 and 2) is clearly clock regulated, a functional ncRNA will only arise with the coincidence of the initiating miRNA. This scenario explains a mechanism for very specific regulation of *ARF *transcript degradation that is possibly dependent on both internal and external cues [59].

While few snoRNAs were detected by the arrays, one such ncRNA, *snoRNA77 *(At5g10572), cycled with a peak expression in the late evening (data not shown). This class of snoRNA is believed to target certain transcripts for chemical modification, namely 2'-O-methylation [60]. Circadian clock regulation of these transcripts suggests that this form of transcriptional modification could, in part, be circadian regulated as well. However, behavior of this transcript was arrhythmic when measured using QRT-PCR of two independent time courses (data not shown). The irreproducibility could be due to a false positive in the tiling array data and analysis or the QRT-PCR data, or due to experimental differences between time courses.

### Circadian clock regulation of natural antisense transcripts

Perhaps one of the more uniquely revealing aspects of a genome tiling array is the ability to differentiate probe strandedness. Indeed, rhythmic NATs were detected for 7% (n = 1,712) of the protein coding genes detected by the arrays (Table S4 in Additional data file 4). Among them were the core clock associated MYB transcription factors *LHY *and *CCA1*, and the *PSEUDO RESPONSE REGULATORS *(*TOC1*, *PRR3*, *5*, *7*, and *9*) (Figures 1, 2, 3, and 4). On the other hand, no NATs were observed for *GI*, *LUX*, or *ELF3*. Among the aforementioned rhythmic NATs, all exhibited a similar time of peak expression as the sense transcript. Overall, the majority of the rhythmic NATs overlapped with circadian regulated sense transcripts with a similar phase of expression (Figure 5d). The expected outcome of NAT expression based on functional characterization and expression pattern of the *Neurospora *core clock gene *FREQUENCY *[61] is inverse expression of the complementary transcript. This leaves in question the potential role of the circadian regulated NATs we detected with similar expression to their corresponding sense transcripts. The use of reverse transcriptase to generate the array probe has been shown to generate artifacts in the form of fragments antisense to coding sequences presumably derived from self priming or mispriming by other fragments [62,63]. This bias, if real, would have to be sequence specific, or it would be ubiquitous across genes, which we do not see. Considering splice junctions are not palindromic, NATs spliced in a similar fashion to sense transcripts, and exhibiting nearly identical expression patterns, are generally artifacts. At the same time, extensive anti-correlated expression of *cis*-NAT pairs resulting in subsequent siRNA has been observed in *Arabidopsis*, but this is only a trend and many do not adhere to this rule [27,64,65]. As with miRNA, observations at the whole genome level without genetic experimentation might not resolve a complex relationship between sense and antisense pairs. However, consistent with the detection of rhythmic introns in otherwise arrhythmic genes, we detected 813 instances of rhythmic *cis*-NATs with an arrhythmic corresponding sense strand transcript (Table S6 in Additional data file 4). In these examples, there was obviously no anti-correlated sense strand pattern resolved, and the absence of a circadian-regulated coding transcript argues against the NATs as experimental artifacts, as do the nearly 8,000 NATs detected by Stolc *et al*[66] that exhibited greater hybridization intensity on the antisense strand than the sense strand in *Arabidopsis *cell cultures. The overall phase distribution of the NATs, regardless of sense strand cycling, was clearly distinct from the coding transcript phase distribution mentioned earlier (Figure 5a). Rather than an overrepresentation of rhythmic transcripts just prior to dawn and dusk, NATs, as with rhythmic sense strand introns (Figure 5c), are enriched towards the morning.

### Circadian clock regulation of intergenic regions

Numerous regions (n = 1,052) not annotated as expressed portions of the genome in TAIR7 exhibited circadian behavior (Tables S7 in Additional data file 4). These areas consist of several different classes. The first are simple annotation errors, where the array hybridization implies a larger transcript than that found in the annotation. Criteria to identify this type are that they are immediately adjacent features to the annotated transcript with a similar phase of expression, such as PRR3 and FKF1, which have three and two cycling intergenic features that would extend the annotation of the 3' end by at least 147 bp each (Figures 2d and 4a). A second class of cycling intergenic regions has supportive expressed sequence tag evidence that is not incorporated into the formal annotation. These include protein coding transcripts as well as ncRNAs [67]. Perhaps the most interesting regions are those with scant or no support from expressed sequence tags or previous tiling array efforts [14,66]. For example, a region of at least 350 bp on chromosome 5 (6,839,029 bp to 6,839,383 bp) is rhythmic, and a coding or functional noncoding transcript is not evident (Figure 6d).

## Conclusions

Numerous forms of ncRNA are well known to be an integral part of genomes, yet many of these transcripts, described here and by others, detected by tiling arrays in several organisms fail to qualify as a functionally characterized ncRNA type [8]. Genome-wide transcription studies have forced a new paradigm of genome organization where most of the genome is expressed, yet often with an unknown function (see, for example, [68]). In addition to documenting the existence of such transcripts, we have described a very specific rhythmic expression behavior that is likely controlled by only a small number of genes making up the *Arabidopsis *circadian clock [31]. The patterns within this study alone strongly suggest these are meaningful expression patterns. For example, antisense transcripts often exhibited very different expression patterns from sense strand transcripts. Also, genes classified as pseudogenes/transposons are severely underrepresented among circadian regulated transcripts, both on sense and antisense strands. Thus, mechanisms of clock regulation were either not maintained with loss of gene function or did not spontaneously occur, suggesting that the novel rhythmic transcription described within is functional.

## Materials and methods

### Plant materials and sample preparation

Seedlings of *Arabidopsis thaliana *accession Col-0 were grown on MS media (supplemented with 2% D-glucose and solidified with 1% agar) 7 days in 12 h light:12 h dark cycles under white fluorescent bulbs at 100 μmol m^-2 ^s^-1 ^before release to constant light and temperature. Samples were collected every 4 h beginning at the time of lights on, ZT0. RNA was extracted by using the Qiagen (Valencia, CA, USA) RNeasy Plant Mini Kit. Labeled cRNA probes were synthesized according to standard Affymetrix (Santa Clara, CA, USA) protocol.

### Array design and annotation

We used high-density oligonucleotide GeneChip^® ^*Arabidopsis *Tiling 1.0R and 1.0F arrays. Each array is composed of more than 3.2 million 25-bp perfect match features along with corresponding mismatch features of either the Watson (1.0F) or Crick (1.0R) sequence strand. On average, each probe was spaced every 35 bp of genome sequence. As previously described [69], perfect match probes from the *Arabidopsis *Tiling 1.0F array were megablasted against the *Arabidopsis *genome release version 7 (TAIR7) [30] including mitochondria and chloroplast sequences with word size ≥ 8 and E-value ≤ 0.01. Single perfect matches, without a second partial match of >18/25 bp, were selected, giving a total of 1,683,620 unique features. These were mapped to annotated mRNAs as intron, exon, inter-genic region, or flanking probes that span an annotated boundary. Background correction and quantile normalization were performed separately on the forward and reverse strand arrays using the affy Bioconductor package in R according to Bolstad *et al*[70]. The Affymetrix AtTILE1 Genechip data (.CEL files) have been deposited at the Gene Expression Omnibus [GEO:GSE13814].

### Fourier/spectral analysis

Hybridization efficiencies of oligonucleotide probes on tiling arrays vary considerably and some probes tend to be unresponsive. Thus, to avoid spurious decreases of signal in the spectral analysis from poorly responsive probes, we filtered out probes that are lowly expressed (mean <3) and furthermore show very little variation (standard deviation < 0.25) across the time series, leaving a total of 1,609,258 features between both the forward and reverse strand arrays. The 12 measurements for each probe were standardized and Fourier analysis was used to evaluate the RNA expression pattern over the 2-day time course [71]. To exploit redundancy of features, we grouped all probes for the same exon based on the TAIR7 genome annotation [30], or applied 200-bp windows centered on each intronic or intergenic probe position while stopping at exon boundaries. We then computed the 24-hour spectral power F24 from the average of the standardized probes within a group, following Wijnen *et al*[71]. To assess the significance of these F24 scores, we built empirical null distributions that take into account the number of probes (weight) that went into the calculation of the spectral power. The family of null distributions was calibrated from the distribution of scores of all probes annotated as intergenic. We parametrized these distributions as exponential functions, which gave excellent fits (Additional data file 6). The *p*-values for all features were then computed from the fitted distributions. The labeling method, which used oligo dT for first strand amplification of the RNA, produces 3' biased probes; therefore, any annotation unit with at least two features satisfying *p *< 0.005 was considered circadian regulated. For Figure 2, the phases for genes were computed from the circular averages of the phase in individual exons using CIRCSTAT [72].

## Abbreviations

NAT: natural antisense transcript; miRNA: microRNA; ncRNA: noncoding RNA; QRT-PCR: quantitative reverse transcriptase PCR; siRNA: short interfering RNA; snoRNA: small nucleolar RNA; TAIR: The *Arabidopsis *Information Resource.

## Authors' contributions

SPH and SAK conceived the study. SPH and JMG carried out the experiments. FN, TQ, JOB, and SPH analyzed the data. SPH, FN, JOB, and SAK drafted the manuscript. JOB, HC, and JRE and carried out the array annotation and web interface support. All authors read and approved the final manuscript.

## Additional data files

The following additional data are available with the online version of this paper. Additional data files 1 and 2 are tables listing the spectral analysis of each microarray time course. Additional data file 3 is a figure comparing the spectral analysis of a gene array time course with the tiling array time course. Additional data file 4 is a series of tables extracted from the spectral analysis. Additional data file 5 is a series of figures demonstrating experimental verification of observations made with the tiling arrays. Additional data file 6 is a figure of the distributions of the exponential functions from the spectral analysis.

## Supplementary Material

Additional data file 1 Spectral analysis of each microarray time course. Click here for file

Additional data file 2 Spectral analysis of each microarray time course. Click here for file

Additional data file 3 Spectral analysis of a gene array time course with the tiling array time course. Click here for file

Additional data file 4 Supplementary tables on the spectral analysis. Click here for file

Additional data file 5 Experimental verification of observations made with the tiling arrays. Click here for file

Additional data file 6 Distributions of the exponential functions from the spectral analysis. Click here for file
