# Supplementary figures and images for: Exploring the transcriptional landscape of plant circadian rhythms using genome tiling arrays
Source: Genome Biol. 2009 Feb 11;10(2):R17. doi: 10.1186/gb-2009-10-2-r17 (PMC2688271; doi:10.1186/gb-2009-10-2-r17)

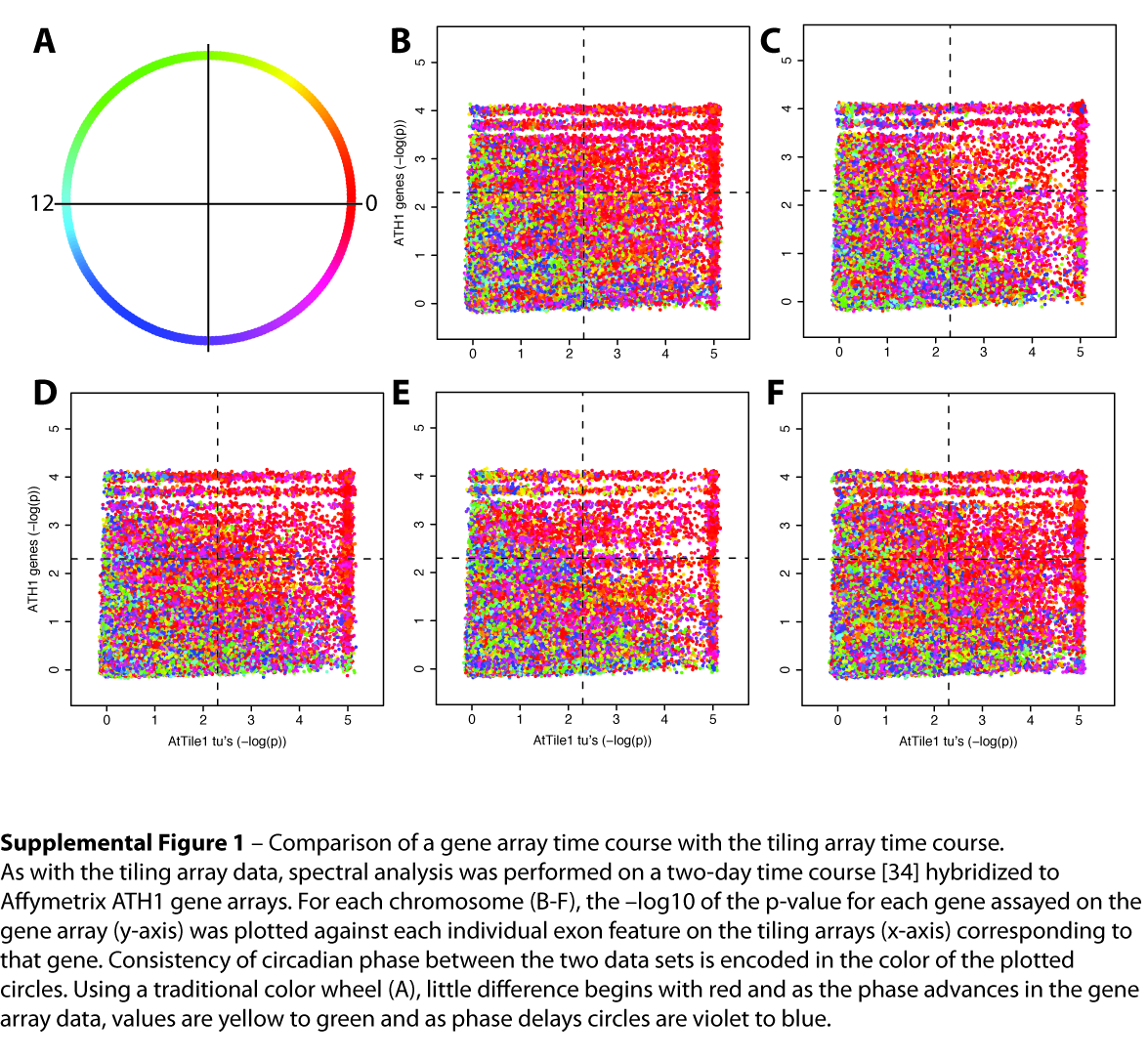

Supplement: Additional data file 3 — Spectral analysis of a gene array time course with the tiling array time course. [file gb-2009-10-2-r17-S3.tiff]

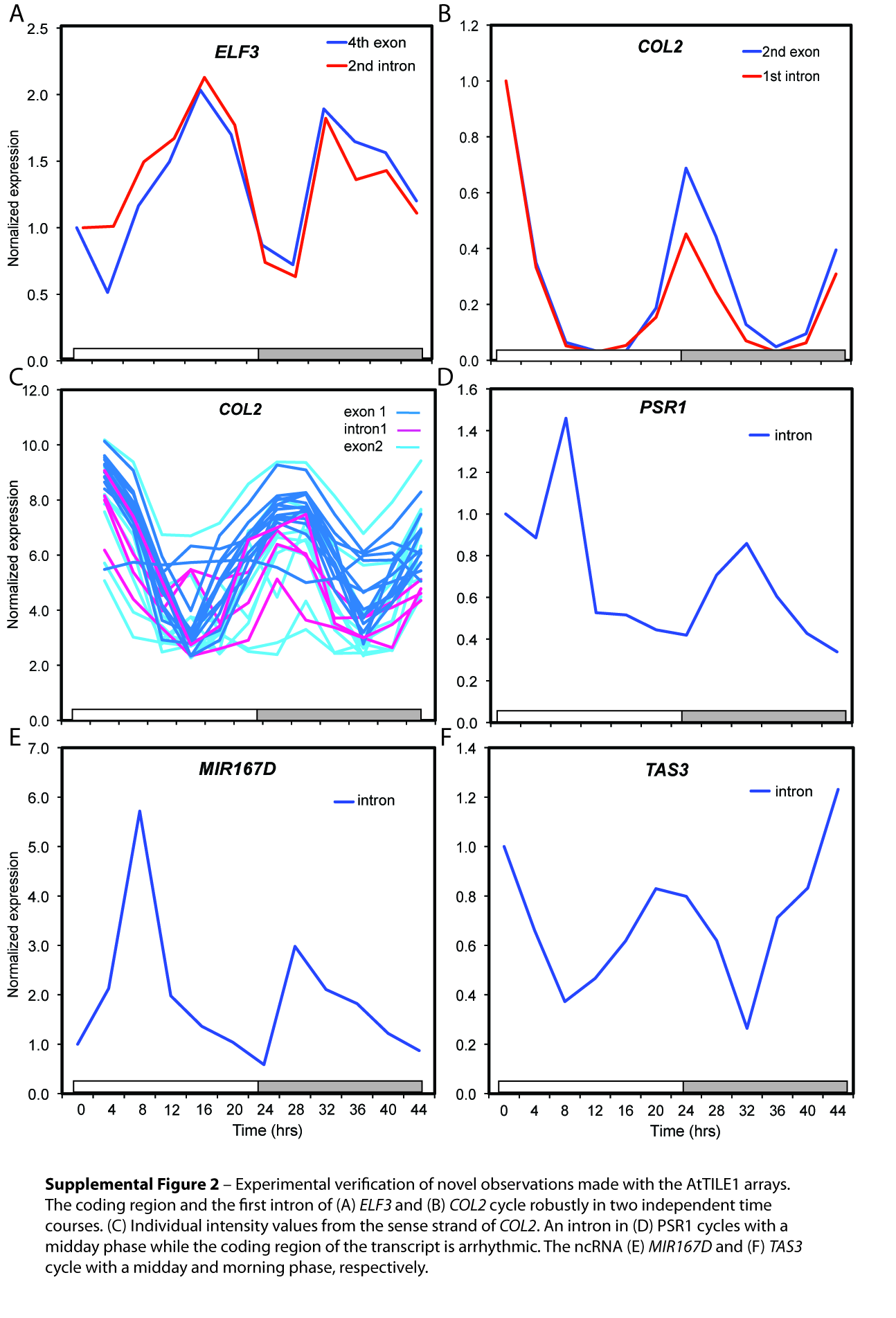

Supplement: Additional data file 5 — Experimental verification of observations made with the tiling arrays. [file gb-2009-10-2-r17-S5.tiff]

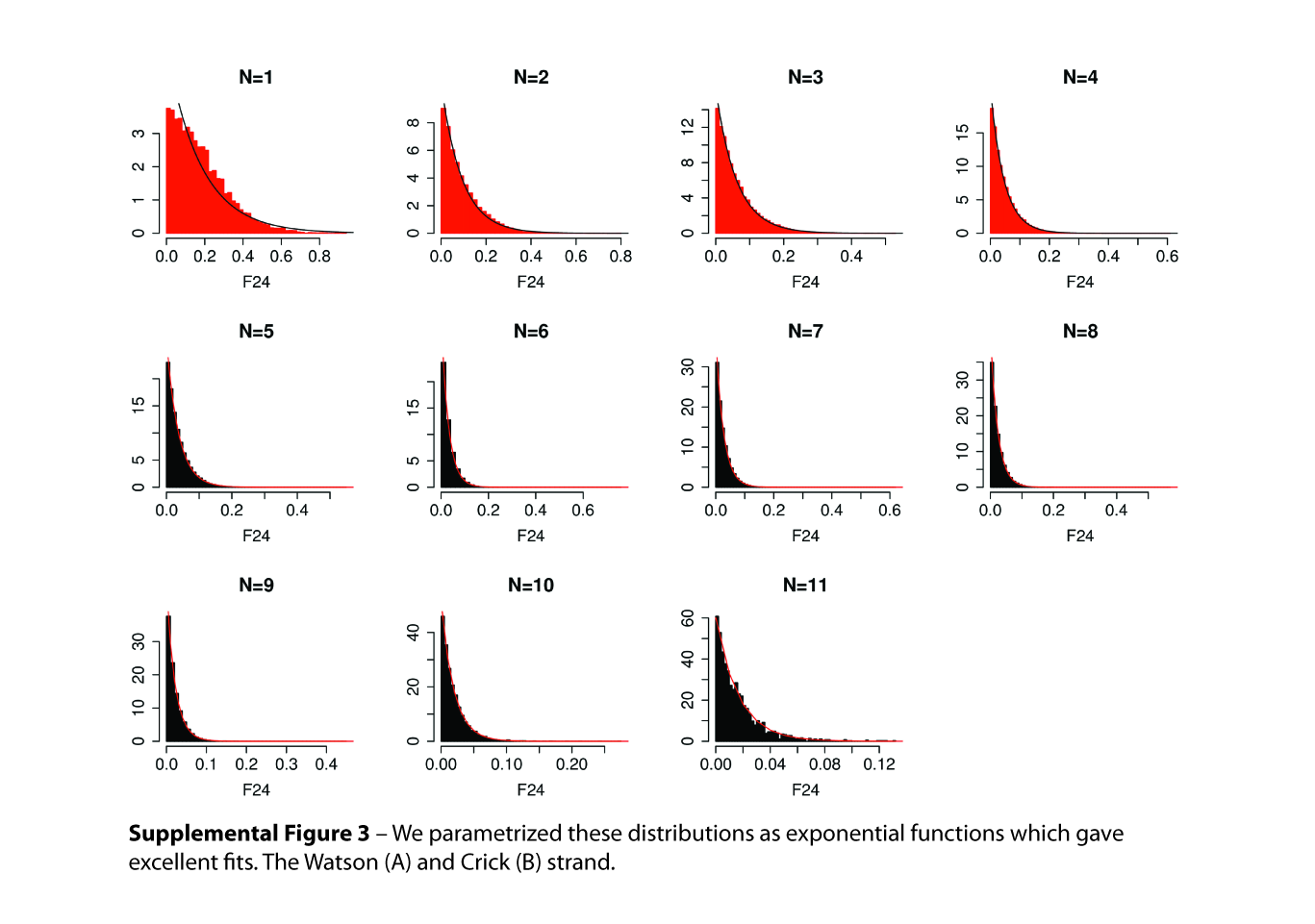

Supplement: Additional data file 6 — Distributions of the exponential functions from the spectral analysis. [file gb-2009-10-2-r17-S6.tiff]
